# Molecular mechanisms and breeding strategies for heat tolerance in vegetable crops under global warming

**DOI:** 10.1093/hr/uhaf309

**Published:** 2025-11-06

**Authors:** Yanlong Li, Xi Zhang, Chan Xia, Ting Wu, Yuyu Gao, Lingen Zeng, Zhuoxuan Wu, Xiongze Dai, Fang Yuan, Feng Liu, Sha Yang, Xuexiao Zou

**Affiliations:** Engineering Research Center for Horticultural Crop Germplasm Creation and New Variety Breeding, Ministry of Education, Key Laboratory for Vegetable Biology of Hunan Province, College of Horticulture, Hunan Agricultural University, Changsha, Hunan 410128, China; Engineering Research Center for Horticultural Crop Germplasm Creation and New Variety Breeding, Ministry of Education, Key Laboratory for Vegetable Biology of Hunan Province, College of Horticulture, Hunan Agricultural University, Changsha, Hunan 410128, China; Engineering Research Center for Horticultural Crop Germplasm Creation and New Variety Breeding, Ministry of Education, Key Laboratory for Vegetable Biology of Hunan Province, College of Horticulture, Hunan Agricultural University, Changsha, Hunan 410128, China; Engineering Research Center for Horticultural Crop Germplasm Creation and New Variety Breeding, Ministry of Education, Key Laboratory for Vegetable Biology of Hunan Province, College of Horticulture, Hunan Agricultural University, Changsha, Hunan 410128, China; Engineering Research Center for Horticultural Crop Germplasm Creation and New Variety Breeding, Ministry of Education, Key Laboratory for Vegetable Biology of Hunan Province, College of Horticulture, Hunan Agricultural University, Changsha, Hunan 410128, China; Engineering Research Center for Horticultural Crop Germplasm Creation and New Variety Breeding, Ministry of Education, Key Laboratory for Vegetable Biology of Hunan Province, College of Horticulture, Hunan Agricultural University, Changsha, Hunan 410128, China; Engineering Research Center for Horticultural Crop Germplasm Creation and New Variety Breeding, Ministry of Education, Key Laboratory for Vegetable Biology of Hunan Province, College of Horticulture, Hunan Agricultural University, Changsha, Hunan 410128, China; Engineering Research Center for Horticultural Crop Germplasm Creation and New Variety Breeding, Ministry of Education, Key Laboratory for Vegetable Biology of Hunan Province, College of Horticulture, Hunan Agricultural University, Changsha, Hunan 410128, China; Engineering Research Center for Horticultural Crop Germplasm Creation and New Variety Breeding, Ministry of Education, Key Laboratory for Vegetable Biology of Hunan Province, College of Horticulture, Hunan Agricultural University, Changsha, Hunan 410128, China; Engineering Research Center for Horticultural Crop Germplasm Creation and New Variety Breeding, Ministry of Education, Key Laboratory for Vegetable Biology of Hunan Province, College of Horticulture, Hunan Agricultural University, Changsha, Hunan 410128, China; Engineering Research Center for Horticultural Crop Germplasm Creation and New Variety Breeding, Ministry of Education, Key Laboratory for Vegetable Biology of Hunan Province, College of Horticulture, Hunan Agricultural University, Changsha, Hunan 410128, China; Engineering Research Center for Horticultural Crop Germplasm Creation and New Variety Breeding, Ministry of Education, Key Laboratory for Vegetable Biology of Hunan Province, College of Horticulture, Hunan Agricultural University, Changsha, Hunan 410128, China

## Abstract

Extreme heat driven by climate change poses a catastrophic threat to global vegetable production, undermining nutritional security because of the heightened physiological sensitivity and succulent tissues of these crops. This review synthesizes the multistage impacts of heat stress across critical developmental phases—from germination to reproduction—emphasizing morphological impairments (such as leaf wilting and floral abortion) and physiological disruptions (including photosynthetic inhibition and oxidative damage). We systematically dissect thermotolerance mechanisms in vegetables, highlighting transcriptional reprogramming by HSFs, WRKY, and NAC transcription factors; chaperone-mediated proteostasis via HSPs; epigenetic remodeling; Ca^2+^-ROS signaling pathways; and the role of phase separation dynamics. Importantly, we propose six strategic pathways to develop heat-resilient vegetables: harnessing natural variation through pan-genome-driven allele mining; employing biotechnological interventions such as CRISPR-mediated editing and synthetic promoters; engineering multistress tolerance by targeting conserved ‘core response’ pathways; exploiting epigenetic memory to achieve transgenerational resilience; optimizing source-sink dynamics with ‘’Climate-Responsive Carbon Optimization; and applying plant growth regulators and nanotechnology to enhance thermotolerance. Together, these strategies chart a clear roadmap for climate-smart vegetable breeding and call for interdisciplinary collaboration to translate molecular discoveries into practical breeding approaches for sustainable food systems under escalating thermal extremes.

## Introduction

Vegetables are indispensable components of a balanced human diet, providing essential nutrients such as dietary fiber, vitamins, and minerals that support critical physiological functions—from enhancing digestive health to reducing the risk of chronic diseases like cardiovascular disorders and diabetes [1]. Despite their nutritional significance, the impact of environmental changes on nutritionally important (nonstaple) vegetables has received relatively little attention, even though these crops appear to be relatively sensitive to environmental shifts [2, 3]. For example, a 4°C increase above a 20°C baseline reduces the average yields of vegetables and legumes by 31.5%, highlighting the serious threat that temperature extremes pose to food security and agricultural sustainability [2]. These trends underscore the urgent need to develop climate-resilient strategies to safeguard vegetable productivity in a warming world.

Plants utilize two principal thermal adaptation mechanisms in response to environmental temperature fluctuations. The first is thermomorphogenesis, a developmental reprogramming triggered by moderate warming, which leads to morphological changes such as hypocotyl elongation and flowering time adjustment, thereby enhancing plant fitness [4]. The second is the heat stress (HS) response, which is activated under acute thermal shock and can result in catastrophic damages, including growth arrest, reproductive failure, and cellular dysfunction [5, 6]. Thermomorphogenesis is orchestrated by the coordinated action of sensors (e.g. phytochrome B), integrators [e.g. *Phytochrome interacting factor 4* (*PIF4*)], and downstream effector genes [7, 8]. In contrast, HS responses are initiated via membrane-bound thermosensors and intracellular detection of protein denaturation or changes in membrane fluidity, leading to activation of signaling cascades involving calcium ions (Ca^2+^) and hormones such as abscisic acid and ethylene [6, 9, 10]. These pathways converge on master regulators like heat shock transcription factors (HSFs), which transcriptionally activate molecular chaperones (e.g. HSP70, HSP90) to refold denatured proteins and alleviate proteotoxic stress [11, 12]. Subsequently, plants regulate the expression of heat-responsive genes through multilayered mechanisms, including post-transcriptional regulation by noncoding RNAs (such as miR398-mediated mRNA cleavage) [13], epigenetic regulation through histone modifications (e.g. acetylation) and DNA methylation that alter chromatin accessibility [14, 15], as well as post-translational modifications (such as phosphorylation of HSFs) that dynamically modulate protein activity and stability, among others [6, 16]. While these mechanisms have been extensively elucidated in the model plant *Arabidopsis thaliana* and rice (*Oryza sativa*), research on heat adaptation mechanisms in vegetable crops remains fragmented. Notably, most studies on vegetables have focused on tomato (*Solanum lycopersicum*), leaving significant gaps in our understanding of species-specific adaptation strategies across diverse vegetable crops.

This review synthesizes current knowledge on HS impacts spanning critical growth stages of vegetable crops—from germination to reproduction—and elucidates vegetable molecular mechanisms underpinning thermotolerance, including Ca^2+^-reactive oxygen species (ROS) crosstalk, HSF-HSP regulatory hierarchies, and epigenetic dynamics. We systematically evaluate conventional and emerging genomic-driven breeding strategies—from marker-assisted selection to CRISPR/Cas9-mediated genome editing—and propose system-integrated approaches to accelerate the development of climate-resilient varieties. By bridging mechanistic insights from model systems with translational applications in agronomy, this work aims to catalyze targeted innovations that safeguard vegetable productivity against escalating climatic volatility.

## Plant thermosensing mechanisms: from receptor recognition to physiological responses

Plants have evolved multifaceted thermosensing strategies to perceive elevated temperatures and initiate appropriate developmental or stress-responsive programs. These mechanisms span both extracellular and intracellular levels, incorporating membrane-based sensors, intracellular thermoregulatory proteins, and downstream signaling pathways that culminate in physiological changes enhancing heat adaptation [6].

### Membrane-associated thermosensors and signal transduction

The plasma membrane is a primary site for temperature perception due to its high thermosensitivity. Heat-induced increases in membrane fluidity alter the conformation and activity of embedded proteins, triggering early signaling events. For example, cyclic nucleotide-gated channels (CNGCs) and annexins (e.g. ANN1) mediate Ca^2+^ influx in response to thermal stimuli, initiating Ca^2+^-dependent signaling cascades [17, 18]. The Ca^2+^-ROS feedback loop, involving *Respiratory Burst Oxidase Homolog D* (*RBOHD)* and *ANN1*, generates self-propagating electrical and chemical waves that amplify thermal perception [19, 20]. Additionally, lipid-derived messengers such as phosphatidic acid and inositol polyphosphates, produced by Phospholipase D and Phospholipase C/Diacylglycerol Kinase pathways, modulate membrane dynamics and protein activity, contributing to thermosensation [21].

### Photoreceptor-mediated thermosensing

Canonical light receptors like phytochrome B (phyB) also act as thermosensors. Under warm conditions, phyB undergoes thermal reversion from its active Pfr form to the inactive Pr form, releasing repression of *PIF4* and *PIF7*, which are two bHLH transcription factors (TFs) that activate thermomorphogenesis, including hypocotyl elongation, leaf hyponasty, and early flowering [22–24].

Recent evidence suggests that intracellular proteins also contribute to temperature perception through phase separation mechanisms. For instance, EARLY FLOWERING 3 (ELF3), a circadian component with a prion-like domain (PrLD), undergoes liquid–liquid phase separation at elevated temperatures, forming condensates that sequester ELF3 away from the *PIF4* promoter, thereby enhancing *PIF4* expression [25]. Similarly, Thermo- with ABA-response 1 (TWA1), an intrinsically disordered transcriptional coregulator, has been identified as a temperature sensor essential for both basal and acquired thermotolerance in *A. thaliana* [26]. Upon heat exposure, TWA1 undergoes conformational changes and accumulates in nuclear subdomains, where it interacts with JAM TFs and corepressors Topless (TPL)–Topless-related (TPR) to form a heat-induced repressive complex. This complex regulates early heat-responsive genes such as *HSFA2*, contributing to downstream thermoprotective responses [26].

### RNA- and chromatin-level thermosensing

Temperature fluctuations also modulate RNA secondary structures and chromatin accessibility, affecting gene expression. For example, high temperatures promote hairpin formation in the 5^**′**^ UTR of *PIF7* mRNA, facilitating its translation and supporting thermomorphogenic growth [27]. Epigenetically, the histone variant H2A.Z, which represses gene expression by stabilizing nucleosomes, is depleted from chromatin at elevated temperatures, enhancing the transcription of HS-responsive genes such as *HSFA2* and *FLOWERING LOCUS T* (*FT*) [28–30].

### Protein translocation and spatial thermosignaling

Thermal cues also induce subcellular relocalization of specific proteins, modulating gene expression via spatial signaling. The glycolytic enzyme glyceraldehyde-3-phosphate dehydrogenase (GAPC), e.g. translocates from the cytoplasm to the nucleus under HS, where it activates Nuclear factor Y subunit C10 (NF-YC10), initiating transcription of heat-responsive genes [31]. Similarly, proteins such as OsNTL3 and Thermotolerance 3.1 (TT3.1) undergo membrane-to-nucleus or endosomal translocation upon heat exposure, linking membrane signal perception with intracellular responses [32, 33].

### Physiological phenotypic outputs

These diverse thermosensing mechanisms converge on a suite of physiological and developmental responses that enhance plant survival under elevated temperatures. Under moderate warming, plants undergo thermomorphogenesis, including hypocotyl elongation, petiole expansion, and early flowering, which help dissipate heat and optimize development [22–24]. In contrast, acute HS activates the heat shock response, marked by rapid induction of heat shock proteins to refold denatured proteins, preserve proteostasis, and maintain cellular integrity. Thermal cues also integrate with hormonal signaling pathways such as abscisic acid, ethylene, and auxin, which regulate stomatal closure, root architecture, and reproductive success under HS, ultimately contributing to thermotolerance and yield protection in crops [6].

## Climate resilience in food systems

**Figure 1:**
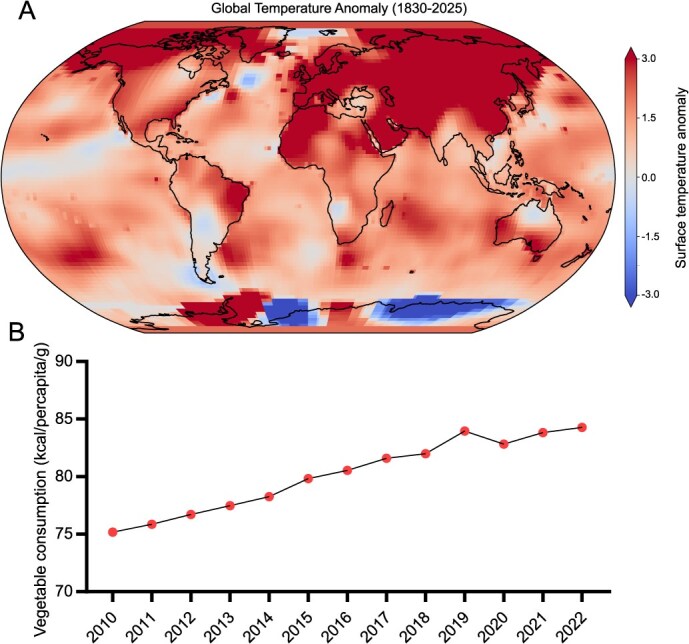
Global surface temperature anomalies and trends in vegetable consumption. (A) This color-coded Robinson projection world map illustrates the progression of global surface temperature anomalies，based on the 30-year baseline period from 1951 to 1980. Areas with higher-than-normal, lower-than-normal, and near-normal temperatures are differentiated according to the legend. The map frame specifically shows global temperature anomalies for the year 2025. (B) Statistical data of global vegetable consumption from 2010 to 2022.

Compared to two centuries ago, temperatures across most regions of the world have risen by ~2.65°F (Fig. 1A) (Sourced from NASA’s dataset), accompanied by increasingly frequent episodes of extreme heat [34]. These changes have imposed severe negative impacts on crop production, with high-moisture and heat-sensitive vegetable crops facing particularly elevated risks [2, 3]. Despite the significantly higher vulnerability of vegetables to climate change compared to staple cereals, current research on climate adaptation remains predominantly focused on rice, maize (*Zea mays*), and wheat (*Triticum aestivum*) [35, 36], leaving a conspicuous gap in systematic attention to vegetables. Anthropogenic greenhouse gas emissions drive a continuous rise in global surface temperatures, leading to more frequent and intense heat waves that significantly impair crop productivity [36, 37]. Staple cereals are highly sensitive to temperature increases, with models indicating that a 1°C rise could reduce yields of wheat, maize, rice, and soybean (*Glycine max*) by 6.0%, 7.4%, 3.2%, and 3.1% respectively [38]. In stark contrast, vegetable crops often demonstrate even greater vulnerability to heat stress. For instance, potato (*Solanum tuberosum*) yields may decline by up to 35% when heat and drought stress are applied during the flowering stage. This reduction was observed in a controlled pot experiment where six cultivars were exposed to day/night temperatures of 35°C/25°C for 2 weeks [39]. Similarly, tomato is highly sensitive to elevated temperatures, especially during reproductive development. In a controlled study, exposure to 42°C–45°C severely reduced fruit set and yield in heat-sensitive genotypes, with yield reductions exceeding 95% compared to tolerant lines [40]. In sweet pepper (*Capsicum annuum*), high temperatures (29°C/23°C) significantly impacted fruit set and yield in sensitive genotypes. In a growth chamber study, yield per plant dropped from 2703 to 66.6 g in the most susceptible genotype, indicating a yield difference of over 97% between genotypes [40]. Likewise, chili pepper genotypes showed strong variation in yield under tropical heat stress conditions. In a comparative study, the most heat-tolerant genotype yielded 1144 g/plant, while the most sensitive yielded only 102 g/plant under day temperatures reaching 38.5°C–39.3°C, suggesting a yield disparity of ~91% [40]. It is important to note that these results of vegetables were obtained under controlled or semicontrolled experimental conditions, and actual yield losses in open-field agriculture may vary depending on stress duration, cultivar, and management practices. Projections further warn that by 2050, processing tomato yields in major global producers (e.g. USA, Italy, China) could decline by 6% due to warming, with potential shifts in primary production areas driven by changing climate dynamics [41]. Concurrently, rising global per capita vegetable consumption (increasing from 75.18 kcal/per capita/g in 2010 to 84.28 kcal/per capita/g in 2022) heightens supply chain fragility (Fig. 1B) (reported by the FAO) [42]. For example, the UK now sources 32% of its vegetable imports from climate high-risk zones (a substantial increase from 20% in 1987), exposing it to significant price volatility and nutritional shortfalls during extreme weather events [43]. In 2023, extreme high temperatures resulted in a 10%–30% yield loss of fruit and vegetable crops in various regions [44]. Such regional declines not only directly impact local food security but also risk triggering a domino effect across the global horticulture industry through shifting climate zones and increased frequency of extreme weather events. Therefore, bridging the critical research gap in vegetable heat adaptation is urgently needed to address this escalating threat to global food security.

## Multistage impacts of high temperature on vegetables

Vegetable species exhibit distinct thermal optima and thresholds, which profoundly shape their growth trajectories and productivity. While moderate temperature increases within optimal ranges (e.g. 20°C–25°C for most species) can accelerate development, exceeding species-specific tolerance limits triggers irreversiblex damage [39, 45] (Table 1). Temperature-related limitations affect every developmental phase, from seed germination through reproductive maturation, ultimately influencing both vegetable crop quality and economic returns.

**Table 1:** The effect of high temperature stress on physiological attributes of some vegetables

**Vegetable**	**Temperature**	**Impact stage**	**Effects**
Tomato	34°C	Reproductive development	Reduced pollen viability and appearance of anthers with pistil-like structures [46]
Tomato	36°C	Vegetative development	Decreased chlorophyll contents and CO_2_ assimilation rate [47]
Potato	25°C	Vegetative growth	Reduced the rate of photosynthesis and carotenoid content [48]
Pepper	33°C	Reproductive development	Reduced pollen viability and fruit set [49, 50]
Pepper	42°C	Vegetative development	Decreased chlorophyll contents [51]
Leafy radish	40°C	Vegetative growth	Changed stomatal characteristics and decreased photosynthetic rate [52]
Radish	40°C	Vegetative growth	Reduced chlorophyll contents [53]
Water spinach	42°C	Vegetative growth	Decreased chlorophyll content, photosynthetic rate, carbon fixation, and increased respiration rate [54]
Sweet potato	37°C	Vegetative growth	Reduced chlorophyll content and storage root-flesh color [55]
Lettuce	25°C	Germination	Reduced germination, tip burn [56]
Okra	42°C	Germination, vegetative growth, reproductive development	Poor germination, flower drop, inferior pod quality, increase in fiber and pectin contents of the pod [57]
Pea	38°C	Vegetative growth	Reduced chlorophyll and carotenoid contents [58]
Cabbage	40°C	Vegetative growth	Delayed growth, yellowing and wilting of leaves, and in severe cases, destruction of the formation of leaf balls and an increase in susceptibility to infectious disease [59]
Carrot	35°C	Germination	Seed germination may be erratic or reduced [60]
Chickpea	40°C	Vegetative growth	Decreased the content of chlorophyll [61]
Chickpea	35°C	Reproductive stage	Reduced pollen production per flower, pollen germination, pod set, and seed number [62]
Broccoli	30°C	Reproductive development	Uneven-sized flower buds on broccoli inflorescences [63]
Cucumber	35°C	Vegetative growth	Leaves sunburn, growth retardation of stems and roots, fruit miscreation, and plant death, which harshly affects cucumber yield and fruit quality [64]

### Seed germination

HS negatively impacts seed germination by reducing both success rate and the speed of seedling emergence [65]. Elevated temperatures disrupt critical cellular processes and impair the metabolic activities necessary for seed activation, leading to delayed germination, decreased seedling vigor, and reduced overall viability [66, 67]. Additionally, HS can cause structural damage to the seed coat, creating a physical barrier that hinders radicle emergence [68]. The optimal temperature range for germination of most vegetable seeds typically falls between 20°C and 25°C. As temperatures rise above this optimal range, germination rates steadily decline [4]. In lettuce (*Lactuca sativa*), for instance, germination responds to a temperature change of as little as 1°C, highlighting the extreme sensitivity of some vegetable species to thermal variation [4]. At ~40°C, most vegetable seeds reach their maximum thermal tolerance limit, beyond which germination can be severely inhibited or completely halted [69, 70] (Table 1).

Significant reductions in germination rates have been reported under HS conditions for various vegetable crops, including okra (*Abelmoschus esculentus*) [71], onion (*Allium cepa*) [72], spinach (*Spinacia oleracea*) [73], carrot (*Daucus carota*) [74], tomato [75], and pepper [76]. Specifically, tomato seeds have an optimal germination temperature range of 24°C–28°C. Germination rates and seedling vigor decrease significantly at temperatures >28.5°C; at 31.5°C, seed germination drops to ~50%, and no germination occurs at 36°C [75]. Similarly, okra seed germination is entirely inhibited at 40°C [71]. For cool-season vegetables such as radish (*Raphanus sativus*) and spinach, an increase in temperature from 30°C to 35°C results in substantial declines in germination rates across various radish genotypes [73, 77]. Similarly, celery (*Apium graveolens*) thrives best at 15°C–20°C, and most cultivars fail to grow properly when temperatures exceed 26°C, reflecting its high sensitivity to heat during both germination and vegetative growth [78]. Therefore, comprehensive morphological, physiological, and molecular studies are required to better understand the impact of HS on vegetable seed germination to facilitate the identification of specific genes contributing to heat tolerance during the seed germination stage.

### Nutritional growth

HS significantly alters the morphology of vegetable plants. Elevated temperatures inhibit longitudinal growth, reducing plant height. Leaves, as primary photosynthetic organs, display marked changes: reduced leaf area, increased thickness, and occasional curling [73, 79]. HS also negatively affects branching, diminishing both branch number and overall photosynthetic capacity. These morphological disruptions impair growth, development, and yield [80].

In leafy vegetables such as cabbage (*Brassica oleracea* var. *capitata*) and lettuce, HS causes cellular-level structural damage, disrupts physiological functions, accelerates chlorophyll degradation and leaf whitening, reduces water content, increases cell wall lignification, and creates brittle, paper-like leaves [73]. Cauliflower (*B. oleracea* var. *botrytis*) and broccoli (*B. oleracea* var. *italica*) are highly sensitive to HS; elevated temperatures disturb hormonal balance and carbon-nitrogen metabolism, leading to excessive vegetative elongation, abnormal head formation, and disrupted pigment synthesis [81]. In cucumber (*Cucumis sativus*), HS impairs cell elongation and division, shortens fruit length, and reduces both water balance and dry matter accumulation, resulting in lighter fruits [82]. In potato, HS disrupts assimilate transport to tubers, delays development, and induces physiological disorders such as black heart, diminishing both quality and commercial value [64]. Overall, high temperatures accelerate development, shorten vegetative phases, and limit accumulation of photosynthetic resources, leading to incomplete reproductive organ development and significant yield reductions.

### Impact on flowering and fruiting

High temperatures severely affect flowering and fruiting in vegetables, typically reducing fertility through impaired reproductive organ development [83]. Plant sexual reproduction encompasses gametophyte (pollen and ovule) development, pollination and fertilization, and postfertilization seed and fruit formation, with male gametophytes (pollen grains) being particularly sensitive to HS [84, 85]. HS reduces pollen quantity, alters morphology, impairs cell wall integrity, and disrupts metabolic activity, leading to decreased viability and fertilization efficiency [86]. As a result, vegetable crops frequently exhibit extensive flower and fruit drop under high-temperature conditions, while abnormal fruit development leads to irregular shapes, inconsistent sizes, and heightened disease susceptibility [87–89]. In broccoli, floral development is highly temperature dependent: e.g. the cultivar Green Harmony F1 ceases floral progression at the inflorescence meristem stage under 28°C or at the floral primordium stage under 22°C, leading to the formation of a cauliflower-like or intermediate curd, instead of a normal broccoli head produced at 16°C [90]. Under prolonged exposure to 32°C, the pollen viability of tomato decreased 4-fold compared to the control [91]. In tomatoes, photosynthetic capacity is impaired at ~30°C, and flowering and fruiting are inhibited at 35°C, with widespread flower and fruit abscission occurring above 40°C. Reduced pollen viability limits pollination and fertilization success, contributing to substantial yield losses [92]. High temperatures during fruit maturation also impair quality; for instance, lycopene synthesis in tomatoes slows above 30°C and is substantially hindered above 35°C, resulting in uneven fruit coloration and diminished market value [93]. Similar reproductive impairments have been observed in chickpeas (*Cicer arietinum*) and common beans (*Phaseolus vulgaris*) under HS [94], further highlighting the critical importance of flowering and fruiting periods in the evaluation of heat tolerance in vegetable crops.

### Systemic consequences

Beyond direct physiological damage, high temperature stress accelerates plant development, shortens the vegetative phase, and limits photosynthate accumulation [95–97]. This results in undersized reproductive organs, incomplete seed development, and physiological disorders such as black heart in potatoes. Collectively, these multistage impacts underscore the urgent need for targeted interventions to mitigate thermal vulnerabilities throughout the vegetable production chain.

## Impact of high temperature on vegetable physiology

### Impact of high temperature on photosynthesis, gas exchange, and respiration in vegetables

HS imposes profound effects on the photosynthetic efficiency, gas exchange, and respiratory metabolism of vegetable crops [98]. At the cellular level, elevated temperatures disrupt key components of the photosynthetic apparatus, including the thylakoid membrane, photosystem I (PSI), PSII, cytochrome b6f complex, electron transport chain, ATP synthesis, and carbon fixation pathways, ultimately inhibiting overall photosynthetic efficiency [99–101]. Among these, PSII is particularly heat sensitive [102]; for instance, cabbage exposed to temperatures above 32°C displays decreased maximum quantum yields, indicating PSII impairment [103]. Similar heat-induced reductions in PSII activity have been observed in spinach due to aggregation of the light-harvesting complex II and in potato, where PSII photochemical efficiency drops by 88% after 12–20 hours of HS [104, 105]. In heat-sensitive cucumber genotypes, marked alterations in PSII function occur above 40°C [106], and tomato plants exposed to 40°C show significant declines in PSII performance [107]. Additionally, HS accelerates the degradation of photosynthetic pigments such as chlorophyll and carotenoids, which further limits photosynthetic activity, as seen in chilies [108], tomatoes [107], and okra [109].

The activity of the key photosynthetic enzyme Rubisco is also compromised under HS. Moderate HS significantly reduces Rubisco activity, which in turn limits stomatal conductance and photosynthetic rates in many vegetable species. Reduced Rubisco isoform expression under HS has been documented in tomato [110], pea (*Pisum sativum*) [111], spinach [112], and potato [113]. Moreover, HS disrupts the integrity of cellular membranes—including plasma, mitochondrial, chloroplast, and thylakoid membranes—through protein denaturation and changes in fatty acid composition. In cauliflower, such disruptions lead to lower pigment concentrations, reduced chlorophyll fluorescence, and impaired photosystem performance [114].

Stomatal conductance, a key regulator of CO_2_ influx and transpiration, is also significantly affected by HS. High temperatures typically induce stomatal closure, restricting CO_2_ uptake and further reducing photosynthetic rates. Meanwhile, changes in stomatal behavior impact transpiration: daytime HS increases transpiration rates, leading to plant water deficits and reduced water potential. High temperatures also alter vapor pressure gradients and reduce plant hydraulic conductivity, limiting water supply to leaves. The ability to maintain adequate gas exchange and CO_2_ assimilation is closely linked to heat tolerance. For example, in tomato plants acclimated to 32°C, reduced net CO_2_ uptake is closely associated with decreased stomatal conductance, underscoring the sensitivity of tomato to temperatures above 30°C [115].

Respiratory metabolism is also affected in complex ways by HS. Initially, high temperatures can increase respiration rates, rapidly consuming photosynthetic products. However, prolonged heat exposure leads to cellular damage and subsequent respiratory dysfunction. For instance, elevated night temperatures enhance respiration in peas, resulting in reduced sugar content and compromised market quality [116]. In tomatoes, higher night respiration rates at 32/26°C have also been observed [117]. Due to limited research on HS-induced respiratory changes in vegetables, increased photosynthetic rates are often used as indirect indicators for identifying heat-tolerant genotypes.

### Cell membrane stability and protective enzyme systems

Under HS, vegetable plants accumulate ROS, such as superoxide anions, hydrogen peroxide, singlet oxygen, and hydroxyl radicals (OH) [118]. These ROS target essential cellular components, including lipids, proteins, polysaccharides, RNA, and DNA, leading to oxidative stress and membrane damage—particularly through lipid peroxidation and protein degradation. HS also increases membrane fluidity due to higher fatty acid unsaturation, resulting in electrolyte leakage and structural damage to cell membranes [119].

To counteract ROS, plants activate antioxidant defense mechanisms, including enzymatic antioxidants [such as superoxide dismutase (SOD), peroxidase (POD), and catalase (CAT)] and nonenzymatic antioxidants (such as vitamins C and E, and flavonoids) [120]. However, when HS intensity exceeds the plants’ threshold tolerance, these defense systems become overwhelmed, intensifying oxidative damage. In tomato anthers, exposure to 36°C/25°C (day/night) induces ROS accumulation and structural abnormalities [93]. Electrolyte leakage from leaf tissues is often used as an indicator of membrane stability under HS [121]. For example, heat-tolerant tomato lines exhibit lower electrolyte leakage compared with susceptible lines [122]. In chilies, plants harboring susceptible genotypes exposed to 35.6°C display higher cellular membrane damage and electrolyte leakage [108]. In cucumber, lower electrolyte leakage correlates with greater heat tolerance at 40°C [106]. Symbiotic associations with endophytic fungi can also mitigate membrane lipid desaturation and enhance cucumber heat tolerance [123].

### Water physiology

HS disrupts water physiology in vegetables by affecting water uptake, transport, and transpiration. Elevated temperatures decrease root water absorption capacity and increase leaf transpiration rates, leading to water deficits and symptoms such as wilting. In tomatoes, HS disturbs leaf water status and reduces root hydraulic conductivity [124]. In potatoes, gradual exposure to HS reduces internal water content, damages photosynthetic pigments, and decreases gas exchange characteristics [125]. These disruptions in plant water relations adversely affect cell structure, turgor pressure, and various physiological processes, ultimately threatening the survival and productivity of vegetable crops.

## Molecular response mechanisms of vegetables under high-temperature stress

**Figure 2:**
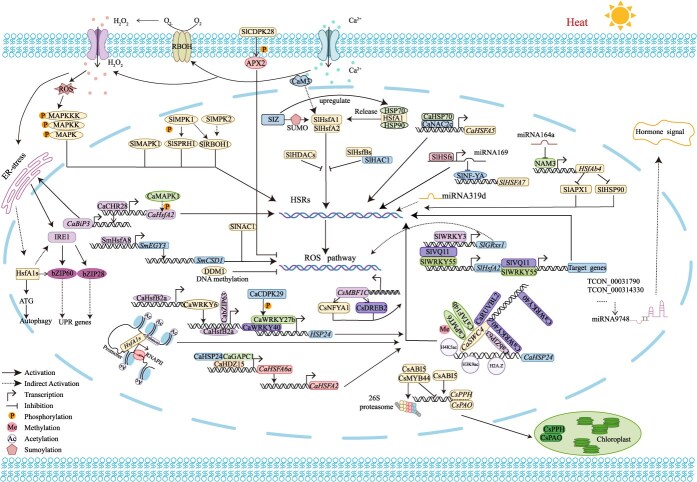
Integrated molecular network governing thermotolerance in vegetable crops. This schematic depicts the sophisticated multitiered regulatory network underlying heat stress (HS) adaptation in vegetable crops, centered on hierarchically organized HSFs. Key transcriptional cascades include HSF-WRKY-NAC modules—such as *CaWRKY40*-*CabZIP63* mutual reinforcement, *SlWRKY55*-*SlVQ11* activation of *SlHsfA2*, and CaNAC2c nuclear translocation activating *CaHSFA5*—alongside epigenetic control via chromatin remodelers (CaSWC4, CaCHR28) and histone modifiers (HAC1, CaPMT6) that deposit active marks (H2A.Z, H3K9ac) to rapidly induce effector genes (*CaHSP24*, *CaBiP3*). Post-transcriptional fine-tuning occurs through heat-responsive small RNAs (e.g. *miR169* derepressing NF-YA, *miR164a* targeting *NAM3*-*HSFA4b*) and lncRNAs, while protein homeostasis is maintained by chaperone interactions (CaHSP70-2 stabilizing CaHDZ15/CaGAPC1), SUMOylation (SlSIZ1 enhancing HsfA1 activity), and ER-UPR pathways (bZIP28/60). Cross-talk with calcium signaling (CaM3), kinase networks (CPK28 targeting APX2; MAPKs as negative regulators), and hormone responses further integrates stress perception, collectively optimizing ROS scavenging, photosynthetic integrity, and cellular protection under HS.

Understanding the molecular mechanisms underlying vegetable responses to high-temperature stress is crucial for developing heat-resilient cultivars. Recent studies have identified a variety of genetic, transcriptional, and signaling networks that coordinate thermotolerance in vegetables. We provide a summary of the key molecular strategies and pathways involved in HS adaptation, serving as a visual framework for the detailed mechanisms discussed below (Fig. 2).

### The function of HSFs in HS response in vegetables

HSFs serve as master regulators of plant thermotolerance, orchestrating transcriptional reprogramming during HS. Under nonstress conditions, HSF proteins remain inactive through complex formation with molecular chaperones—predominantly HSP70 and HSP90—in the cytoplasm or nucleus. Upon HS exposure, protein denaturation triggers chaperone dissociation, liberating HSFs that translocate to the nucleus where they bind heat shock elements (HSEs) in target gene promoters to initiate transcription.

Diverse HSF genes have been characterized across vegetable crops and classified into classes A, B, and C based on oligomerization domains. Species-specific inventories include tomato (24 genes), potato (27 genes) [126], chili pepper (25 genes) [127], carrot (35 genes) [128], peanut (*Arachis hypogaea*; 46 genes) [129], and eggplant (*Solanum melongena*; 20 genes) [130]. These evolutionarily conserved regulators mediate critical thermotolerance mechanisms: potato *StHSF004/7/14/19* coordinate heat shock responses [126], while eggplant *SmeHsfA5* and *SmeHsfA6a* show marked upregulation under thermal stress [121, 130]. In eggplants, SmHSFA8 interacts with the SmEGY3 protein, enhances its transcriptional activation of *SmCSD1*, promotes hydrogen peroxide (H_2_O_2_) accumulation, and thereby contributes to the regulation of plant heat tolerance [131]. In chili pepper, *CaHsfA1d*, *CaHsfA2*, and *CaHsfA3* exhibit pronounced HS-induced expression among 17 *CaHsfA* genes [127]. Functional validation via *CaHsfA1d* silencing in pepper and heterologous overexpression in *Arabidopsis* confirmed its role in thermotolerance through H_2_O_2_ homeostasis maintenance [132]. Tomato HSF networks—particularly *HsfA1a*, *HsfA2*, and *HsfB1* [133, 134]—exemplify sophisticated regulatory hierarchies. HS rapidly upregulates *HsfA1a*, which directly activates *HsfA2* and *HsfB1* transcription to induce heat-tolerant genes [135]. HsfA1a and HsfA2 form heterodimers that synergistically amplify downstream expression, whereas HsfB1 fine-tunes responses through competitive promoter binding with HsfA1a or recruitment of histone deacetylases (HAC1) to suppress gene overexpression [135, 136]. Crucially, *HsfA1* functions as a hierarchical regulator analogous to its *Arabidopsis* counterpart [137]. Its activation involves cytoplasmic retention via HSP70/90 complexes under basal conditions [138]; HS-triggered complex dissociation enables nuclear import where post-translational modifications (e.g. phosphorylation, SUMOylation) enhance transactivation capacity. The SIZ1 SUMO E3 ligase (SlSIZ1) directly mediates HsfA1 SUMOylation to potentiate activity and independently upregulates HSP70 protein level, thereby conferring thermotolerance through dual mechanisms in tomato [139].

### The function of other TFs in HS response

Beyond core HSFs, additional TF families—including WRKY, NAC, MYB, bZIP, DREB, and HD-ZIP—participate in complex regulatory networks governing vegetable thermotolerance. These TFs establish multilayered heat-defense mechanisms through intrafamily cooperation, interfamily synergy, and signaling pathway crosstalk. WRKY TFs critically regulate plant HS responses [140]. In tomato, *SlWRKY3* positively modulates thermotolerance; its overexpression reduces ROS accumulation by directly binding promoters of *Glutaredoxin S1* (*SlGRXS1*) clusters encoding ROS-scavenging proteins [141]. SlWRKY55 activates *SlHsfA2* transcription via promoter binding, facilitated by SlVQ11 interaction. Both SlWRKY55 and VQ motif-containing protein 11 (SlVQ11) enhance *SlHsfA2* transcriptional activity through physical association [142]. In pepper, *CaWRKY40* promotes thermotolerance by activating W-box-containing promoters to suppress ROS and cell death. CaWRKY6 transcriptionally activates *CaWRKY40* [143], while CaHsfB2a modulates both *CaWRKY6* and *CaWRKY40* expression under HS [144]. CaWRKY40 further interacts with phosphorylated CaWRKY27b, which acts as a transcriptional coactivator to enhance CaWRKY40’s DNA-binding affinity and transactivation of thermotolerance genes (e.g. *CaHSP24*), despite CaWRKY27b itself lacking direct promoter-binding capacity due to its WRKYGMK motif [145]. In nonheading Chinese cabbage, BcWRKY23 enhances thermotolerance by activating *BcWRKY25* and downstream PAL genes (*BcPAL1/2*), which elevate PAL activity under HS. Under prolonged HS, accumulated BcVQ11A interacts with BcWRKY23/25 to suppress PAL activation, forming a feedback loop that modulates thermotolerance [146]. NAC TFs contribute significantly to vegetable thermotolerance [147, 148]. Tomato *SlNAC1* deficiency reduces HSP/antioxidant accumulation, elevating ROS and compromising HS resilience [147]. In pepper, CaNAC2c exhibits nucleocytoplasmic shuttling: under nonstress conditions, CaHSP70 sequesters it cytoplasmically, whereas HS triggers nuclear colocalization, stabilizing CaNAC2c and enhancing DNA-binding to activate heat-responsive genes *CaHSFA5* [148]. R2R3-MYB TFs widely mediate abiotic stress adaptation. Overexpression of tomato *Lycopersicon esculentum Anthocyanin 2* (*LeAN2*) (anthocyanin biosynthesis regulator) elevates photosynthetic rates, PSII efficiency, and nonenzymatic antioxidant activity while reducing ROS under HS [107]. In cucumber, HS co-induces *Abscisic Acid Insensitive 5* (*ABI5*) and *MYB44*. ABI5 promotes chlorophyll degradation by activating *Pheophytinase* (*PPH)* and *Pheophorbide a Oxygenase* (*PAO*), while MYB44 inhibits this process. ABI5-MYB44 interaction disrupts MYB44 binding to *PPH*/*PAO* promoters, accelerating MYB44 degradation and chlorophyll breakdown [149]. bZIP TFs coordinate essential HS responses. Among the tomato’s 26 bZIP genes, *SlbZIP10*, *SlbZIP32*, and *SlbZIP33* show significant HS upregulation in leaves and roots [150]. HS-induced misfolded proteins activate endoplasmic reticulum-unfolded protein response (ER-UPR) via *bZIP28*/*bZIP60*, whose expression is *HsfA1a*-dependent. These TFs cooperatively enhance ER-phagy through ATG gene induction in tomatoes [151]. Pepper CabZIP63 activates *CaWRKY40* transcription, establishing a mutual reinforcement loop with *CaWRKY40* to amplify thermotolerance [152]. Cucumber CsbZIP2 binds the *miRNA9748* promoter, enhancing HS resilience through *miRNA9748*-mediated cleavage of *CsNPF4.4* alongside jasmonic acid (JA) signaling [153]. DREB TFs govern abiotic stress adaptation. Tomato SlDREBA4 regulates HSP genes and phytohormone biosynthesis pathways under HS [144, 154]. CaHDZ15 interacts with both CaHsp70-2 and CaGAPC1. CaHsp70-2 protects CaHDZ15 and CaGAPC1 from 26S proteasome-mediated degradation via physical interactions, while CaGAPC1 enhances CaHDZ15’s DNA-binding affinity to the *CaHSFA6a* promoter. This cooperative action amplifies *CaHSFA6a* transcription, initiating a transcriptional cascade (*CaHDZ15* → *CaHSFA6a* → *CaHSFA2*) essential for thermotolerance [155]. In cucumber, HS induces *Multiprotein Bridging Factor 1c* (*CsMBF1c*) expression. Overexpression enhances thermotolerance, while *Csmbf1c* mutants exhibit sensitivity. *CsMBF1c* stabilizes photosynthetic machinery under HS and coordinates heat-related TFs and ER protein processing genes. It physically interacts with CsDREB2 and CsNFYA1, with *CsNFYA1* overexpression in *Arabidopsis* confirming its thermotolerance role. CsMBF1c elevates *CsNFYA1* transcriptional activation, establishing a regulatory module for cucumber heat adaptation [156].

### Function of HSPs in HS response

HSPs, essential guardians of cellular homeostasis, constitute a conserved class of molecular chaperones critical for plant thermotolerance. Classified by molecular weight into HSP100, HSP90, HSP70, HSP60, and small HSP families, their primary functions encompass facilitating protein folding and repair, maintaining structural stability, and mediating intracellular protein trafficking [157]. Elevated HSP accumulation under HS is well-documented across vegetable species: in tomato, HSPs persist poststress, indicating sustained protective roles; in pepper, transcripts of *CaHSP70*, *CaHSP60*, *CaHSP20*, and *CaHSP16.4* are markedly induced by high temperatures [105, 158–160]; in lettuce, spatiotemporal induction of HSP70 modulates thermotolerance through calmodulin interaction, suppressing heat-induced premature bolting [73, 161]; in legumes, common beans exhibit coordinated expression of *HSP70* and heat shock TF *PsHSFA* [162]; and in cabbage, thermotolerant lines show enhanced *BoHsp70* expression [163]. Notably, BiP3, a specialized HSP family member, confers thermotolerance in *Solanaceous* crops (pepper and tomato) by increasing chlorophyll retention, reducing ROS accumulation and electrolyte leakage, upregulating defense genes, and mitigating endoplasmic reticulum (ER) stress. This process is governed by a conserved *CaCHR28*-*CaHsfA2*-*BiP3* regulatory axis, wherein both chromatin remodeling and transcriptional activation converge to promote *BiP3* expression under HS [164].

In-depth mechanistic studies have revealed that HSP-mediated thermotolerance relies on a highly coordinated “recognition-binding–transfer-refolding/solubilization” cascade. This involves functional specialization among HSP subfamilies: HSP40 initiates repair by recognizing misfolded proteins, delivering substrates to HSP70, which undergoes ATP hydrolysis-driven conformational cycling to tightly bind and stabilize unfolded polypeptides [165–167]. Under sustained HS, Hop, a TPR-domain protein, bridges HSP70 and HSP90, enabling client transfer and ensuring proper folding of signaling proteins via HSP90’s ATPase activity [168]. For aggregated proteins, HSP100/ClpB collaborates with HSP70 to resolubilize aggregates using ATP hydrolysis [169]. Simultaneously, small HSPs (sHSPs) act as early responders, encapsulating unfolded proteins into stable complexes to prevent irreversible denaturation [170].

In *Arabidopsis*, this network is exemplified by AtHSP70, AtHSP90, and AtHSP101, which respectively prevent denaturation, refold damaged proteins, and resolubilize aggregates, while AtHSP21 protects chloroplast photosystems [171–174]. Cross-species evidence reveals diverse adaptations of this chaperone machinery in crop plants. In rice, the SUMO-conjugating enzyme (SCE1) negatively regulates thermotolerance by repressing HSP24.1 accumulation. The E3 ligase TT1 promotes SCE1 degradation under HS, lowering global SUMOylation and enhancing heat resilience; the *sce1* mutant shows increased HSP abundance and a 15.1% yield increase under field heat [175]. In wheat, TaHSP90.2-B interacts with the nuclear-encoded PSBO protein and facilitates its actin-assisted transport to chloroplasts, PSII assembly. A natural G/A variant in its promoter boosts thermotolerance and increases thousand-grain weight by 8.5% [176]. In tomato, LeHSP21, localized in chloroplasts, protects PSII photochemistry against oxidative damage during HS [177]. In pepper, CaHSP70-2 interacts with CaHDZ15 in an ATP-dependent manner to stabilize its nuclear localization under 37°C. This interaction enhances CaHDZ15-mediated activation of *CaHSFA6a*, which triggers *CaHSFA2*, forming a transcriptional cascade that improves seedling survival under heat. Moreover, CaGAPC1 cooperatively enhances CaHDZ15 DNA-binding capacity, suggesting a regulatory bridge between metabolism and thermotolerance [155]. Collectively, these findings illustrate that the HSP chaperone system is not only evolutionarily conserved but also mechanistically diversified among vegetable and crop species. The core functional modules—substrate recognition, ATP-dependent folding, proteostasis maintenance, and regulatory feedback—are conserved, while their integration with species-specific TFs, signaling molecules, and chromatin regulators reflects adaptive flexibility. This highlights the evolutionary plasticity of HSP-mediated heat responses and provides rich molecular targets for breeding heat-resilient vegetable crops.

### Role of Ca^2+^ in molecular responses to HS

Ca^2+^ serve as pivotal secondary messengers in plant cells, transducing HS signals through rapid cytosolic concentration spikes. These transient elevations generate unique ‘calcium signatures’ decoded by Ca^2+^-binding sensors—including calmodulins (CaMs), calcium-dependent protein kinases (CDPKs)—which activate downstream cascades to modulate gene expression, metabolic adjustments, and physiological adaptations, ultimately enhancing thermotolerance. These calcium-initiated signals further interface with mitogen-activated protein kinase (MAPK) cascades to amplify stress responses [178].

CaM, activated by Ca^2+^ binding, plays a central role in HS perception [179]. Ca^2+^ binding activates CaM3, which plays a key regulatory role in the Ca^2+^-CaM-mediated HS response [180]. In cucumber, HS strongly induces *CsCaM3* expression in the heat-tolerant lines; its overexpression upregulates key HS responsive genes (*CsSOD*, *CsPOD*, *CsCAT*, *CscAPX*, *CsAOX*, *CsHSP70*, *CsHSP90*) and mitigates cellular damage [181]. CDPKs are another important class of Ca^2+^-sensing proteins that are rapidly activated under HS. Upon activation, CDPKs phosphorylate various TFs, thereby modulating the expression of heat-responsive genes. Genome-wide expression studies have shown that multiple CDPK isoforms are markedly induced by HS in various vegetable species. For example, in cucumber, the transcript levels of *CsCDPK2*, *CsCDPK6*, *CsCDPK10*, and *CsCDPK15* increase substantially under elevated temperatures [182]. Similarly, in pepper, nine *CaCDPK* isoforms exhibit significantly enhanced transcript abundance during HS [183]. Notably, CaCDPK29 positively regulates pepper heat tolerance by phosphorylating CaWRKY27b [145]. In tomato, 13 out of 29 identified *CPK* isoforms respond to high-temperature treatment (45°C), with transcripts of *CPK9*, *CPK23*, and *CPK28* accumulating rapidly within the first hour [184]. Tomato *CPK28* (previously named *LeCPK2*) plays an essential role in thermotolerance by targeting *Ascorbate Peroxidase 2* (*APX2*), a key antioxidant enzyme gene, underscoring its potential application in breeding heat-tolerant tomato cultivars for climate change adaptation [185, 186]. MAPK cascades, comprising sequentially activated MAPKKK, MAPKK, and MAPK, transduce HS signals from membrane receptors to cellular targets [187, 188]. In potato, 42°C HS induces *StMPK1* expression, implicating its role in stress response [189]. Tomato MAPKs SlMPK1 and SlMAPK3 act as negative regulators: *SlMPK1* silencing enhances thermotolerance by suppressing SlSPRH1 phosphorylation and preserving antioxidant capacity [190], while *slmapk3* mutants exhibit higher survival rates and reduced oxidative damage [191]. Brassinosteroid signaling enhances thermotolerance by promoting RBOH1-dependent MPK2 activation and apoplastic H_2_O_2_ accumulation, establishing a positive feedback loop among RBOH1, H_2_O_2_, and MPK2 in tomato [192]. Pepper *CaMAPK1* silencing exacerbates HS injury and impairs CaHSFA2/CaHSP70-1 induction [193]. Lettuce *LsMAPK4* knockdown delays heat-accelerated bolting [194]. Integrated Ca^2+^-signaling networks coordinate HS responses through interconnected Ca^2+^-CaM, CDPK, and MAPK modules. These pathways synergize with HSP regulatory networks to enable precise thermal adaptation. Conserved CDPK and MAPK isoforms across vegetable species provide mechanistic insights and theoretical foundations for breeding heat-resilient cultivars.

Beyond classical signaling modules, recent findings highlight the role of protein quality control via autophagy in thermotolerance [195, 196]. Recent studies in tomato revealed that transglutaminase (TGase) enhances thermotolerance by promoting autophagy through stabilization of SAMS2 and stimulation of polyamine biosynthesis, which upregulates ATG genes and facilitates degradation of ubiquitinated proteins under HS [196].

### Role of ROS in vegetable response to HS

HS triggers excessive ROS production—including superoxide (O_2_^−^), H_2_O_2_, and OH—primarily through impaired chloroplast/mitochondrial electron transport chains and respiratory burst oxidase homolog activation [197, 198]. This ROS surge causes lipid peroxidation, protein denaturation, and DNA damage, severely disrupting cellular integrity. To mitigate oxidative stress, plants deploy enzymatic antioxidants (SOD, POD, CAT, APX) and nonenzymatic scavengers (vitamins C/E, carotenoids) that synergistically neutralize ROS [118]. Vegetables employ conserved antioxidant strategies for thermotolerance: cucumber enhances photosynthetic stability and ROS scavenging at 40°C to alleviate heat damage [199]; pepper coordinates reduced ROS accumulation, elevated *P5CS* expression, and increased SOD/POD activities [200]; and tomato upregulates CAT/APX/SOD activities and activates *APX2* transcription via CDPK28 to suppress ROS [186, 201]. Notably, *slmapk3* mutants exhibit reinforced antioxidant capacity, reducing H_2_O_2_ and O_2_^−^ accumulation and enhancing resilience [191]. These findings underscore enzymatic/nonenzymatic ROS regulation as a critical adaptive mechanism in vegetables, providing molecular targets for breeding climate-resilient crops.

### Epigenetic regulation in vegetable HS responses

Epigenetic mechanisms, including DNA methylation, histone modifications, chromatin remodeling, and noncoding RNA regulation, dynamically orchestrate transcriptional reprogramming during HS [202–207]. In tomato, chromatin remodeler Decrease in DNA Methylation 1 (DDM1) maintains genome stability by facilitating DNA methyltransferase activity. The *ddm1b* mutant exhibits enhanced thermotolerance, with elevated fruit/seed set and seedling survival under HS, correlating with differential expression of heat-responsive genes [202, 203]. In broccoli, warming-induced floral abnormalities were attributed to promoter hypermethylation and suppression of apex-expressed genes, which could be reversed by 5-azacytidine, highlighting a direct role of DNA methylation in thermosensitive floral development [90]. Histone modifications further modulate chromatin accessibility: tomato HsfB1 recruits HAC1 to activate heat-responsive genes [136], while *HSFA1a* promotes chromatin looping between enhancers and promoters of targets like *HSFA2* [204]. In cabbage, the H3K36 demethylase Jumonji Domain-containing Protein 18 (BrJMJ18) enhances thermotolerance by modulating flowering and chlorophyll biosynthesis under HS [205]. Pepper employs analogous epigenetic strategies; SWI/SNF Complex Subunit 4 (CaSWC4), as a core chromatin remodeler, orchestrates pepper responses to both HS and pathogen infection by recruiting key TFs (CaWRKY40 and CabZIP63) and depositing active histone marks (H2A.Z, H3K9ac, H4K5ac) at specific target genes, such as *CaHSP24*, thereby promoting their activation. Its activity is enhanced by Putative methyltransferases 6 (CaPMT6)-mediated methylation, which stabilizes CaSWC4 and boosts the enrichment of active histone marks and thermotolerance, while *CaPMT6* silencing impairs these processes. Under stress, CaSWC4 assembles a coactivator complex with RuvB-like 2 (CaRUVBL2) and TATA-box Binding Protein-Associated Factor 14b (CaTAF14b) at stress-specific loci, facilitating chromatin opening and rapid transcriptional reprogramming [206, 207]. Thus, the *CaPMT6* → *CaSWC4* methylation axis and CaSWC4-cofactor chromatin remodeling together form a dual epigenetic mechanism that enables pepper to balance and optimize responses to both biotic and abiotic stresses.

Post-transcriptionally, small RNAs fine-tune HS responses. HSFs activate *miR169* expression, and the resulting *miR169* cleaves the negative regulator *NF-YA*, thereby alleviating its repression on HS effectors like *HSFA7* and forming a thermotolerance-enhancing *HSF*-*miR169*-*NF-YA* feedback loop in tomato [12]. Overexpression of *sha-miR319d* significantly boosts tomato thermotolerance by mitigating heat-induced oxidative damage and enhancing antioxidant defense capacity [208]. *miR164a* stands out as one of the most rapidly responsive small RNAs to HS; it functions by repressing its target *NAM3*. NAM3 acts as a heat-sensitive negative regulator that activates *HSFA4b*, which in turn suppresses key antioxidant gene *APX1* and heat shock protein gene *HSP90* in tomato. Consequently, *miR164a* enhances plant thermoadaptation by modulating ROS scavenging and HSP pathways via the *NAM3*-*HSFA4b* module [209]. Long ncRNAs also contribute significantly: cucumber responds to HS with 2085 differentially expressed lncRNAs (e.g. *TCON_00031790*) that may interact with *miR9748* in hormone signaling pathways [210], paralleled by extensive lncRNA identification in Chinese cabbage [211]. Notably, two lncRNAs from Chinese cabbage have been shown to confer heat and drought tolerance when expressed in *Arabidopsis*, underscoring their functional relevance across species [212]. Collectively, these multilayered regulatory networks enable precise adaptation to thermal stress.

### Phase separation in vegetable responses to HS

Phase separation—a biophysical process driven by weak multivalent interactions (e.g. hydrophobic, electrostatic, cation–π, and π–π interactions)—enables biomolecules to dynamically organize into membraneless biomolecular condensates, such as stress granules (SGs) or nuclear bodies [213]. This mechanism, especially liquid–liquid phase separation (LLPS), is increasingly recognized as a central strategy by which plants regulate HS responses [214–217]. These condensates compartmentalize cellular components without membranes, facilitating signal sensing, transcriptional reprogramming, RNA metabolism, and protein quality control under thermal stress.

Recent studies reveal that phase separation is not merely downstream of stress signaling, but also directly involved in temperature sensing. Photoreceptor phyB, a red/far-red light receptor, undergoes thermal reversion from its active Pfr form to the inactive Pr form under high temperatures. This reversion releases PIF4 and PIF7, which then initiate thermomorphogenesis [22, 23]. ELF3, a circadian clock component with a PrLD, responds to heat by undergoing LLPS, forming nuclear condensates that sequester ELF3 away from the *PIF4* promoter, thereby enhancing *PIF4* expression. [25]. TWA1, an intrinsically disordered transcriptional coregulator, also functions as a temperature sensor. Upon heat exposure, TWA1 undergoes conformational changes, accumulates in nuclear bodies, and interacts with JAM TFs and corepressors TPL/TPR to form a heat-induced repressive complex, regulating early heat-responsive genes such as *HSFA2* [26]. These examples indicate that LLPS enables proteins to act as thermosensors, integrating structural flexibility with environmental responsiveness.

HS frequently induces subcellular relocalization of proteins, often associated with phase transition phenomena: GAPC, a glycolytic enzyme, translocates into the nucleus under HS and activates NF-YC10, a TF essential for HS responses [31]. OsNTL3 and TT3.1 undergo membrane-to-nucleus or endosomal relocalization upon heat exposure, linking spatial dynamics with signal integration [32, 33]. Concurrently, LLPS drives the formation of SGs, which sequester mRNAs and RNA-binding proteins to preserve transcripts and regulate translation under stress. For instance, RNA-Binding Glycine-rich Protein (RBGD2/4) and Acetylation lowers binding affinity (ALBA) proteins undergo LLPS to form SGs that stabilize heat-responsive transcripts, ensuring protective translational control under HS [218, 219].

Although most phase separation studies are conducted in *Arabidopsis*, emerging evidence supports its relevance in vegetable crops. In *Arabidopsis*, MORF8 undergoes solid-like condensation under heat via its Intrinsically Disordered Region (IDR), sequestering RNA editing complexes and suppressing NDH complex mRNA editing, ultimately reducing photosynthetic efficiency [214]. Similarly, RNA-Directed DNA Methylation 16 (RDM16) isoforms (RDL and RDS) cooperatively form nuclear condensates via arginine-rich IDRs, facilitating HSF mRNA splicing and boosting heat tolerance in *Arabidopsis* [215]. FuSang Tree 1 (FUST1), a disordered protein conserved across plant species including tomatoes, responds to elevated temperature through its prion-like domain, initiating SG assembly and promoting thermotolerance [216]. In tomato, the Terminating Flower (TMF) protein senses heat-induced ROS bursts and undergoes LLPS to form transcriptional condensates in the shoot apical meristem, delaying flowering and enhancing thermotolerance via vegetative phase extension [217]. These findings suggest that vegetable crops may utilize conserved LLPS mechanisms to regulate transcription, RNA processing, and developmental transitions during HS. Despite these advances, phase separation research in vegetable crops remains in its infancy. Key challenges include identifying core LLPS-prone proteins in nonmodel crops; dissecting the molecular grammar of IDRs and PrLDs; elucidating cross-talk between phase separation, post-translational modifications, and hormonal pathways; and establishing in vivo visualization and tracking systems to monitor condensate dynamics. Future studies integrating omics, live-cell imaging, and computational modeling will be crucial for mechanistic dissection and crop-level application.

## Future research methods and approaches for vegetable heat tolerance

**Figure 3:**
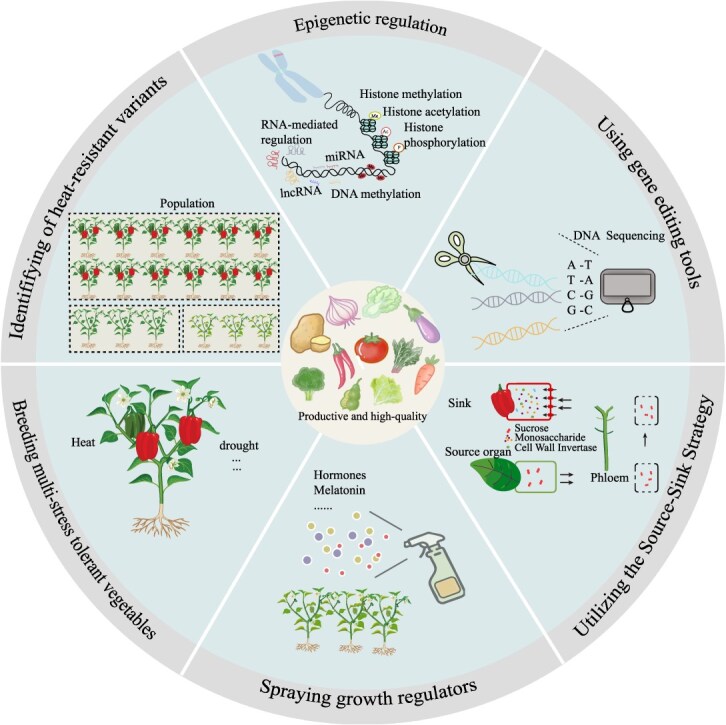
Strategies to mitigate heat stress in vegetable crops. This diagram summarizes six key approaches to overcoming the harmful effects of heat stress in vegetable crops: harnessing natural variation through pan-genome-driven allele mining and breeding heat-tolerant varieties; employing biotechnological interventions such as CRISPR-mediated editing and synthetic promoters to accelerate genetic improvement; engineering multistress tolerance by targeting conserved ‘core response’ pathways to address combined abiotic stresses; exploiting epigenetic memory to achieve transgenerational resilience and enhance thermomemory; optimizing source-sink dynamics via CROCS to support efficient carbon allocation under stress; and applying plant growth regulators and nanotechnology as complementary strategies to improve thermotolerance. Together, these strategies provide a comprehensive roadmap for developing climate-resilient vegetable crops and addressing the challenges posed by global warming.

As global warming accelerates, the development of heat-resilient vegetable crops is paramount for ensuring food security and sustainable agriculture. Recent advances in genetics and biotechnology provide new opportunities to address HS more systematically and effectively. We illustrate six innovative strategies that collectively form a roadmap for enhancing thermotolerance in vegetable crops (Fig. 3).

### Uncovering natural genetic variation to breed heat-resilient vegetables

Exploiting natural genetic variation stands as a transformative strategy for enhancing vegetable thermotolerance. Pioneering work in staple crops has illuminated successful paradigms: Rice *TT1* (encoding a 26S proteasome α2 subunit) harbors allelic variants that accelerate degradation of toxic denatured proteins under HS, while *TT2* (a G-protein γ subunit) exhibits truncating SNPs conferring resilience in tropical japonica rice [220, 221]. Concurrently, *TT3* orchestrates a sophisticated regulatory module wherein chloroplast-targeted E3 ligase TT3.1 ubiquitinates and degrades its negative regulator TT3.2 via multivesicular bodies, mitigating chloroplast damage [67]. Similarly, *QTL on chromosome 12* (*QT12*) modulates grain-quality thermotolerance by disrupting unfolded protein response (UPR)-mediated endosperm homeostasis, with natural variations in *NF-Y* complexes forming an on–off switch for its expression [222]. Beyond cereals, *Arabidopsis ERECTA* allelic variants regulate membrane integrity [223], grapevine *Thermotolerance on chromosome 4* (*TTC4)* haplotypes fine-tune HSP/APX activation via WRKY/SQUAMOSA-promoter binding protein-like 13 (SPL13) interplay [224], and cotton (*Gossypium hirsutum*) *Heat-related Receptor Kinase* (*GhHRK1*) loss-of-function enhances thermotolerance [225]—collectively underscoring the power of QTL/GWAS-driven allele mining.

Despite these advances, research on thermotolerance-associated natural variation in most vegetables remains nascent, with only a few well-characterized QTLs and candidate genes identified so far—significantly fewer than in staple cereals like rice and wheat. In vegetables, notable examples include *qHT1.1* in cucumber (with *Csa1G004990* as a strong candidate gene) and several loci in pepper associated with HSP expression and transcriptional regulation [226, 227]. However, the burgeoning availability of vegetable pan-genomes and high-throughput omics resources now offers unprecedented opportunities to identify favorable variants [228–230]. Moving forward, efforts should prioritize the systematic identification of heat-resilient alleles using integrated genomics and phenomics, as well as the precise deployment of elite haplotypes through marker-assisted pyramiding. Bridging evolutionary genetics with multi-omics profiling will accelerate the development of climate-resilient vegetables, transforming heat tolerance breeding from serendipity to strategic design.

### Biotechnological roadmap for engineering thermotolerant vegetables

The identification of thermotolerance-associated genetic variants necessitates advanced biotechnologies to translate these discoveries into resilient crops. Rapid progress in gene editing and overexpression systems has matured their application in field crops, as exemplified by the targeted manipulation of rice thermotolerance loci: knockout of *TT2* and *TT3.2*, coupled with overexpression of *TT3.1* and *TT1*, significantly enhances field performance under HS [105, 220]. Cross-species expression of *Arabidopsis ERECTA* further demonstrates broad efficacy, boosting thermotolerance in both rice and tomato, suggesting potential for exogenous gene deployment in vegetables [223].

Beyond conventional CRISPR/Cas9, recent advances in precision genome editing have introduced transformative tools such as base editing (BE) and prime editing (PE), which allow single-nucleotide modifications or short sequence insertions without inducing double-stranded breaks (DSBs), thereby improving edit specificity and reducing genomic instability [231, 232]. These systems are particularly promising for fine-tuning heat-responsive promoters or coding sequences in vegetable crops, where allelic diversity is often limited. For example, in tomato, PE was used to insert HSEs into the *Levanase-Insensitive 5* (*LIN5*) promoter, which significantly elevated fruit yield under high-temperature conditions, demonstrating the potential of PE for thermotolerance improvement in vegetable crops [233]. In parallel, the development of novel Cas variants with relaxed PAM constraints—such as SpCas9-NG, xCas9, and SpRY [234–236]—has significantly expanded the editable genome space across diverse plant species, including previously difficult-to-edit vegetable genomes.

A particularly exciting breakthrough is the programmable chromosome engineering (PCE) and RePCE systems, which enable scarless kilobase- to megabase-scale insertions, deletions, inversions, and translocations in both rice and human cells [237]. These systems integrate AI-assisted recombinase engineering (AiCErec) and re-pegRNA strategies to achieve precise structural modifications. For instance, a 315-kb inversion in rice using RePCE conferred herbicide resistance [237], suggesting potential for remodeling large heat-responsive regulatory regions or gene clusters in vegetables. Meanwhile, the AiCE (AI-informed constraints for protein engineering) framework offers a generalizable method for evolving genome editing enzymes. It has been successfully applied to optimize deaminases, nucleases, and reverse transcriptases—resulting in enhanced editors such as enABE8e (narrow window editing) and enSdd6-CBE (high-fidelity BE) [238], which could be deployed for precision editing of HS TFs or epigenetic regulators in vegetable crops. Furthermore, the integration of artificial intelligence (AI) and machine learning tools such as DeepCRISPR, PRIDICT, and TIGER has greatly improved the accuracy of sgRNA design, off-target prediction, and repair outcome modeling [239–241]. These tools are particularly valuable for vegetables with complex genomes or limited genomic resources, as they enable rational target selection and reduce experimental trial time, thereby mitigating transformation bottlenecks.

Synthetic biology approaches also offer transformative solutions; e.g. fusing the heat-inducible *HsfA2* promoter to the chloroplast-targeted *psbA* gene enhances PSII repair and biomass accumulation in multiple crops [242]. Promoter engineering strategies, such as targeted modification of the *Casein Kinase I* (*GhCKI*) promoter in cotton, allow fine-tuned expression of stress-response genes with reduced pleiotropic effects [243]. Despite these advances, the application of CRISPR/Cas and other biotechnology tools in many vegetable species remains hampered by low genetic transformation efficiency, highlighting the urgent need to develop more efficient and genotype-independent transformation protocols. Therefore, complementary approaches such as EMS, T-DNA insertion, and space-induced mutagenesis remain valuable for generating mutant pools. For instance, the *gry3* rice mutant (heat/light-sensitive) and the *pf1* tomato line (parthenocarpic due to *SlHB15A* mutation) exemplify the power of functional genomics in trait discovery [244, 245]. Collectively, integrating next-generation precision editors, AI-guided design, and classical mutagenesis will provide a versatile toolkit for enhancing thermotolerance in vegetable crops, bridging the gap from gene discovery to field deployment.

### Engineering systemic resilience: towards multistress tolerant vegetables

Climate change increasingly exposes crops to concurrent abiotic stresses—termed ‘combined stresses’—that inflict far greater damage than singular threats [246]. Extreme heat and drought, in particular, exhibit intensifying synergy, with global cropland affected by such compound events expanding by 1–2% per decade and causing disproportionate yield losses [247]. Crucially, genotypes that are resilient to single stresses often fail under combined regimens [248, 249], highlighting the urgent need to understand how vegetables respond to such challenges. Recent breakthroughs have revealed sophisticated plant adaptations: e.g. tomato orchestrates organ-specific stomatal regulation, with leaves prioritizing water conservation (by reducing stomatal density and opening under drought), while sepals enhance cooling through temperature-sensitive modulation of transpiration—a divergence mediated by tissue-specific Abscisic acid (ABA) metabolism that persists even after stress is relieved and underscores the necessity of whole-plant phenotyping [250]. In potato, multi-omics profiling under heat, drought, and waterlogging exposures uncovers both unique and shared molecular signatures, such as the universal downregulation of photosynthesis, minor amino acid accumulation, and stress-specific hormone dynamics like ABA induction in drought and waterlogging [251]. Critically, the observation of overlapping transcriptional networks and signal transduction pathways suggests the existence of conserved ‘core response components’ across combined stresses [251, 252]. This calls for a paradigm shift: rather than focusing on isolated stress responses, identifying superior alleles that govern these shared mechanisms will accelerate the breeding of multistress resilient crops. Such an approach must account for adaptation trade-offs, as interventions targeting a single extreme may compromise cross-tolerance. Moving forward, leveraging systems biology and machine learning to decode the universal stress logic will enable the predictive design of vegetables resilient to compounding climate challenges.

### Harnessing epigenetic memory for climate-resilient vegetable breeding

Recent studies in vegetable crops have revealed that epigenetic regulation plays a central role in HS responses, as evidenced by dynamic DNA methylation in tomato *ddm1b* mutants [202, 203], histone acetylation via *HsfB1*-*HAC1* [136], and chromatin remodeling by CaSWC4 in pepper [206, 207]. These findings highlight that vegetable species possess multilayered and responsive epigenetic networks crucial for thermotolerance. However, the potential of these mechanisms to generate heritable stress memory remains underexplored.

Epigenetic mechanisms constitute a sophisticated adaptive framework that orchestrates plant thermotolerance while offering transformative breeding strategies [253]. Under stress, dynamic DNA methylation changes may generate transgenerational memory, enabling progeny to inherit enhanced resilience. For example, stable *Acquired cold tolerance 1* (*ACT1*) promoter hypomethylation in rice confers heritable cold tolerance [254], raising the possibility that similar mechanisms could be harnessed in vegetables. In tomato, *HSFA1a*-mediated chromatin looping enables rapid transcriptional reprogramming during HS [204], suggesting that epigenetically primed chromatin states may underlie memory-like behavior even in vegetables. This memory machinery operates through multilayered circuits: elevated temperatures trigger *HSFA2*-mediated activation of the H3K27me3 demethylase *Relative of Early Flowering 6* (*REF6*), which reciprocally reduces repressive H3K27me3 marks at *HSFA2* loci, establishing a self-reinforcing loop that perpetuates thermal memory [255]. Although this has been characterized in *Arabidopsis*, similar histone mark dynamics have been observed in pepper, where CaSWC4 enriches active acetylation marks (H3K9ac, H4K5ac) at heat-responsive genes, indicating shared regulatory logic [206, 207]. Small RNAs also contribute to heat adaptation: for instance, *miR169*-*NF-YA* feedback loops and *miR164a*-*NAM3*-*HSFA4b* modules in tomato orchestrate post-transcriptional control of ROS and HSP pathways [12, 209]. Meanwhile, lncRNAs such as *TCON_00031790* in cucumber interact with miRNAs in hormone signaling under HS [210]. These examples underscore the rich epigenetic diversity in vegetables and their potential to encode thermal memory. However, direct evidence for transgenerational epigenetic memory in vegetable crops remains limited, and research in this area is still in its infancy—underscoring the need for further mechanistic studies and long-term experimental validation.

Critically, emerging technologies like modular epigenome editing now enable precise manipulation of chromatin states at target loci [256]. Integrating these tools with multi-omics approaches (e.g. ATAC-seq, Hi-C, ChIP-seq) will decode the nexus between chromatin architecture and phenotypic plasticity in vegetables. For future breeding, this paves the way for epigenetic priming, stress imprinting, and nontransgenic thermotolerance enhancement. Developing epigenetic selection markers and heritable chromatin signatures in key vegetable crops will be essential for realizing the full potential of epigenetic memory-based breeding strategies under climate change.

Additionally, although current thermotolerance studies in vegetables primarily focus on annual species, perennial vegetables—such as asparagus (*Asparagus officinalis*) and daylily (*Hemerocallis citrina*)—may possess unique epigenetically mediated adaptation strategies developed over multiple growing seasons. These may include constitutive activation of stress-responsive genes, sustained antioxidant systems, and potential transgenerational memory shaped by repeated thermal exposure. Investigating the epigenetic landscapes of perennial vegetables under HS could uncover novel regulatory elements and chromatin-based thermotolerance mechanisms. Comparative studies between annual and perennial species may thus provide valuable insights for breeding or gene editing efforts aimed at long-term resilience in vegetable crops.

### Optimizing source-sink dynamics: CROCS technology as a climate-adaptive breakthrough for vegetable yield stability

The source-sink paradigm—fundamental to crop productivity since 1928—governs photosynthetic carbon partitioning from source organs (mature leaves) to sink tissues (developing fruits/seeds) via phloem-translocated sucrose [257, 258]. High-temperature stress catastrophically disrupts this equilibrium, as evidenced by supra-additive yield losses (e.g. 31.7% in wheat under combined heat-drought stress) through multitiered mechanisms: impaired photosynthesis reduces sucrose synthesis; diminished vascular bundle capacity hinders transport; and transcriptional reprogramming creates ‘metabolic sink traps’ via downregulated starch biosynthesis genes (*AGPase*, *SS*, *GBSS*) and upregulated degradation genes (*ISA3*, *BAMY2*) [259]. Vegetable systems exhibit parallel vulnerabilities: in sweet pepper, source limitation (shading/leaf pruning) and sink competition (fruit position) synergistically drive floral abortion [260], while tomato suffers blossom drop and erratic fruit development under heat due to suppressed *LIN5* expression—a cell wall invertase governing sucrose partitioning [261].

The Climate-Responsive Carbon Optimization (CROCS) strategy pioneers a transformative solution: precise insertion of a 10-bp heat-responsive cis-element into the endogenous LIN5 promoter dynamically modulates sink capacity. This genetic circuitry enables temperature-adaptive sucrose flux—basal transport at ambient conditions versus enhanced mobilization during heat waves—effectively decoupling yield from climate volatility. Field validation demonstrates robust efficacy: CROCS-engineered tomatoes achieve 14%–47% yield gains under optimal conditions and 26%–33% resilience under HS [233]. By reconciling the conflict between ‘high yield in favorable environments’ and ‘stable production under adversity,’ CROCS exemplifies a paradigm-shifting approach to climate-proof vegetable agriculture through source-sink recalibration.

### Phytohormonal control and the role of growth regulators and nanotechnology in vegetable HS adaptation

**Figure 4:**
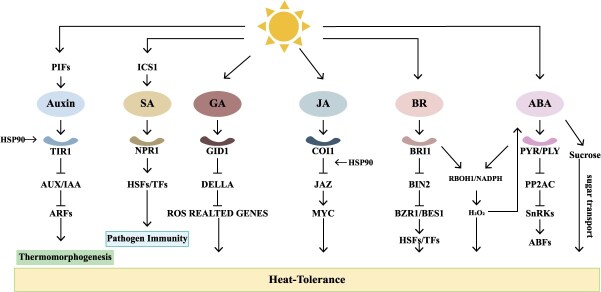
Regulatory mechanisms of hormone biosynthesis and signaling under high temperature. Elevated temperatures modulate plant hormone pathways by either activating or repressing critical molecular nodes. These include enzymes involved in hormone biosynthesis and degradation, hormone transporters, receptor proteins, signal transduction components, and downstream transcriptional regulators. The temperature responsiveness of the auxin pathway has been linked to the phyB-PIF thermosensory module, suggesting a mechanistic connection between thermal perception and hormonal control. However, how other hormone pathways are directly coupled to temperature sensing remains largely unresolved.

Phytohormones play a pivotal role in enabling plants to withstand HS through finely tuned signaling networks (Fig. 4). Understanding key regulatory nodes within these pathways provides promising molecular targets for enhancing thermotolerance.

ABA serves as a central regulator of the HS response, primarily acting through the Pyrabactin Resistance (PYR)/PYR1-Like (PYL) receptor family. Under heat exposure, endogenous ABA levels increase rapidly and transiently [262]. In *Arabidopsis*, ABA binding activates the PYR1-PYL2 receptor complex [263, 264], leading to phosphorylation of downstream TFs such as ABA Responsive Element-Binding Factor (ABF) and AREB via SNF1-Related Protein Kinase (SnRK2.6) [265, 266]. This cascade culminates in the induction of thermotolerance-related genes, including *HSFA6b* [267]. This pathway is conserved across species: in wheat, *TaHsfA6f* overexpression enhances thermotolerance through downregulation of the ABA catabolic gene *CYP707A3* and upregulation of biosynthetic (*AtZEP*) and *APX2* genes. In rice, OsPRMT6b-mediated arginine methylation of the ABA receptor OsPYL/RCAR10 modulates its stability and ABA signaling under heat stress [268]. Similarly, in lettuce, *LsNCED4* upregulation promotes ABA accumulation and improves thermotolerance during seed germination [269]. ABA enhances thermotolerance through multiple mechanisms, including stimulation of apoplastic H_2_O_2_ production via NADPH oxidases (RBOHs), which activates antioxidant defenses. Mutants such as *Atrbohd* show impaired thermotolerance [262]. ABA also regulates carbohydrate metabolism by inducing sucrose transporters and metabolic enzymes, thereby supporting energy homeostasis and signaling under stress [270–272].

Auxin (IAA) modulates heat responses largely through ARF TFs. In rice, high temperature reduces IAA levels, and OsIAA29 competes with OsIAA21 to interact with OsARF17, affecting grain filling [273]. In cucumber, low-dose exogenous IAA improves root activity and reduces ROS under HS, while *CsYUC10b* overexpression enhances thermotolerance via improved photosynthesis and DNA repair [274]. Thermomorphogenic auxin responses involve HSP90-mediated stabilization of Transport Inhibitor Response 1 (TIR1), leading to Aux/IAA degradation and activation of ARFs [275, 276]. PIF4 and PIF7 integrate temperature cues into auxin pathways by regulating *YUCCA8* expression [277, 278].

Brassinosteroids (BRs) enhance thermotolerance via *BRI1*-initiated signaling, involving sequential phosphorylation of Brassinosteroid-signaling kinase 1 (BSK1), Constitutive Differential Growth 1 (CDG1), and BRI1 suppressor 1 (BSU1), inhibition of Brassinosteroid-insensitive 2 (BIN2), and nuclear accumulation of Brassinazole-resistant 1 (BZR1)/Bri1-ems-suppressor 1 (BES1) TFs [279–281]. Thermotolerance depends on BR signaling activation rather than BR concentration. BRs elevate HSP levels, stabilize membrane proteins, enhance translation efficiency [282, 283], and induce ROS signaling through RBOH1 to boost antioxidant activity [284].

JA signaling is mediated by the Coronatine insensitive 1 (COI1)-JAZ module [285]. HS upregulates o*xophytodienoate-reductase 3* (*AtOPR3)*, increasing JA biosynthesis and activating *DREB2A* to promote thermotolerance [286]. Chaperones such as SGT1 and HSP90 maintain COI1 homeostasis and JA signaling efficacy [287]. Warm temperatures induce JA catabolism genes *Jasmonic Acid Oxidase* (*JOX*) and *Sulfotransferase 2A* (*ST2A*) to prevent excessive JA-Ile accumulation and maintain hormonal balance [288].

Salicylic acid (SA) functions mainly through *NPR1*-mediated pathways. SA induces Nonexpresser of Pr Genes 1 (NPR1) nuclear translocation, where it activates defense genes via TGA and WRKY factors [289]. Heat impairs Isochorismate synthase 1-dependent SA biosynthesis in *Arabidopsis*, though application of BTH restores immune function [290]. In rice, heat disrupts GBPL defense-activated condensates (GDAC) body formation and Guanylate-binding protein-like 3 (GBPL3) recruitment to Cam-binding protein 60-like G (CBP60g); overexpression of *CBP60g* or *SAR Deficient 1* (*SARD1*) restores SA signaling and immunity at elevated temperatures [291]. Exogenous SA improves thermotolerance in maize by enhancing antioxidant activity and photosynthetic performance [292]. The DELLA protein family in the gibberellin (GA) signaling pathway plays a key role in plant responses to temperature. DELLA proteins are negative regulators in the GA signaling pathway; they function by binding to the GA receptor GA Insensitive Dwarf1 (GID1), which leads to their degradation [293]. In rice, two alkali-thermal tolerance QTL genes, *Alkali-thermal tolerance 1 (ATT1)* and *ATT2*, have been identified. These genes control gibberellin biosynthesis and regulate the abundance of the Slender Rice1 (SLR1) protein to modulate ROS levels and H3K27me3 deposition in response to alkali-thermal stress. The study also revealed that fine-tuning the level of bioactive gibberellins to a moderate range in rice varieties can minimize yield loss caused by environmental stress [294].

Heat responses are orchestrated through complex crosstalk among hormones, encompassing both synergistic and antagonistic interactions. A notable synergistic mechanism is the BR-ABA-ROS feedback loop, wherein BR-induced H_2_O_2_ promotes ABA biosynthesis, which further amplifies ROS production via mutual induction of RBOHs (‘BR → H_2_O_2_ → ABA → H_2_O_2_’) [295]. Conversely, antagonistic interactions can also occur. In *Setaria viridis*, HS activates ABA, JA, and BR pathways but suppresses IAA, SA, Ethylene, Gibberellin, and Cytokinin signaling, illustrating a strategic reallocation of resources from growth to defense [296].

Given the central role of endogenous phytohormones, their manipulation via exogenous hormone analogs and plant growth regulators (PGRs) represents a promising avenue for improving thermotolerance in vegetable crops. The following section discusses recent advances in PGR and nanotechnology applications for modulating these pathways to enhance heat resilience. The molecular mechanisms governing high-temperature stress responses in crops are not fully elucidated, hampering the development of heat-tolerant vegetable varieties through conventional breeding, which is often slow and costly. As an alternative, PGRs offer an efficient and environmentally sustainable strategy. Exogenous application of ABA, SA, ethylene precursors, and melatonin (MT) has been widely documented to enhance thermotolerance across crops [270, 297, 298]. These compounds act through various mechanisms, including membrane stabilization, enhancement of antioxidant systems, transcriptional regulation of heat-responsive genes (e.g. HSPs and sugar metabolism genes), and improved photosynthetic efficiency. Melatonin, in particular, has emerged as a potent antioxidant and signaling molecule. Both exogenous treatment and genetic engineering (e.g. overexpression of *PlTDC* or *MdASMT9*) have been shown to reduce ROS, protect chloroplast integrity, maintain osmotic balance, modulate ABA signaling to optimize stomatal conductance, and induce autophagy [299–302]. Nanomaterials (NPs) are also gaining traction as tools for enhancing plant stress adaptation. NPs can improve morphological traits, reduce membrane damage, boost antioxidant capacity, and activate heat-responsive genes [303–305]. However, potential risks such as phytotoxicity and environmental impact must be carefully addressed. For instance, under HS, certain NPs like CeO_2_ may intensify stress by inducing ABA/ROS-mediated stomatal closure [306]. Future research should prioritize optimizing PGR and NP application protocols—including timing and concentration—exploring synergistic combinations, and developing precision delivery systems and eco-friendly formulations to sustainably enhance vegetable thermotolerance.

## Conclusions

In the face of accelerating global warming, safeguarding vegetable crop productivity and nutritional security has emerged as a critical challenge for sustainable agriculture and food systems. This review highlights the multifaceted and stage-specific impacts of HS on vegetable growth, development, and quality, underscoring the heightened vulnerability of these crops due to their physiological sensitivity. We have systematically summarized the molecular mechanisms underlying thermotolerance—including intricate regulatory networks involving TFs (such as HSFs, WRKY, NAC, MYB, bZIP, DREB, and HD-ZIP), chaperone-mediated proteostasis, Ca^2+^-ROS signaling, epigenetic remodeling, and emerging roles of phase separation. Furthermore, we have outlined innovative breeding and biotechnological strategies—ranging from pan-genome-driven allele mining and CRISPR-based genome editing to the exploitation of epigenetic memory, optimization of source-sink dynamics, and the application of plant growth regulators and nanotechnology—providing a robust roadmap for developing climate-resilient vegetable varieties. Despite these advances, significant knowledge gaps remain, particularly concerning species-specific adaptation mechanisms and the integration of multistress resilience. Future research should prioritize interdisciplinary collaboration, high-throughput phenotyping, and translational studies to bridge molecular discoveries with practical breeding and agronomic solutions. Ultimately, leveraging these integrative strategies will be essential for ensuring the sustainability and resilience of global vegetable production under escalating thermal extremes.

## Data Availability

This is a review article and does not report any original experimental data. All data and findings discussed herein are derived from previously published studies, which have been properly cited in the manuscript.
